# Chiral Self-Assembly and Chiral Separation of Ext-TEB Molecules on Bi(111)

**DOI:** 10.3390/nano16070399

**Published:** 2026-03-26

**Authors:** Lei Liu, Zheng Wei, Min-Long Tao, Kai Sun, Ming-Xia Shi, Jun-Zhong Wang

**Affiliations:** 1 International Joint Laboratory for Light Alloys (MOE), College of Materials Science and Engineering, Chongqing University, Chongqing 400044, China; 20220901045@stu.cqu.edu.cn; 2 School of Physical Science and Technology, Southwest University, Chongqing 400715, China; taotaole@swu.edu.cn (M.-L.T.); skqtt@swu.edu.cn (K.S.); 3 Key Laboratory for Electronic Materials, College of Electrical Engineering, Northwest Minzu University, Lanzhou 730030, China; 292229061@xbmu.edu.cn

**Keywords:** chiral separation, Ext-TEB, non-edge-sharing honeycomb structure, STM

## Abstract

The two-dimensional chiral self-assembly and chiral separation of achiral Ext-TEB molecules on a Bi(111) surface were investigated using low-temperature scanning tunneling microscopy (LT-STM). At low coverage, the molecules self-assembled into chiral clusters. As the coverage increased, a monolayer film with a non-edge-sharing honeycomb structure was formed. This supramolecular structure exhibited organizational chirality, accompanied by chiral separation. Upon annealing, part of the non-edge-sharing honeycomb structure transformed into a close-packed structure, while retaining the organizational chirality, supramolecular chirality, and pronounced chiral separation. Furthermore, applying a higher bias was found to induce a transition in the electronic state of the non-edge-sharing honeycomb structure, converting it into an edge-sharing honeycomb configuration. This study reveals that the chirality of 1,3,5-tris(4-ethynylphenyl) benzene (Ext-TEB) arises from the rotation of the side-chain phenyl rings, which is induced by the rotation of the molecular axis relative to the substrate lattice. This work presents a strategy for the preparation of chiral nanostructures from achiral molecules due to the spontaneous chiral symmetry generation.

## 1. Introduction

Chirality plays an important role in chemistry, biology, and materials science [1,2,3,4,5,6]. Chirality in nanostructures formed via surface molecular assembly and reaction occurs at the aggregate level. The surface is two-dimensional and provides a symmetry-breaking environment. Chirality can transfer from the molecular level to the aggregate level during assembly or reaction [7,8,9,10]. The understanding of surface chirality processes such as chirality induction, transfer, and amplification has progressed extensively in the past decades [11,12,13]. Besides chiral molecules, achiral molecules can also exhibit chirality through interactions with substrates and associated charge transfer processes. This chirality manifests at different scales: single-molecule chirality (intramolecular structural asymmetry), supramolecular chirality (asymmetry in aggregated multimolecular structures), and organizational chirality (lattice asymmetry in molecular thin films) [14,15,16].

**Figure 1:**
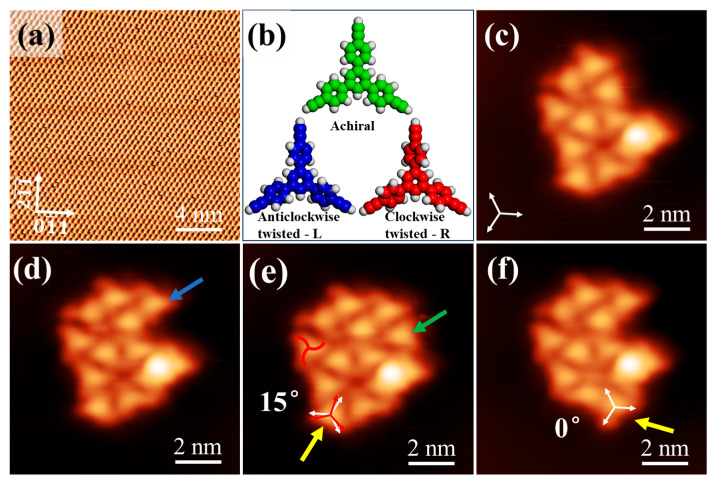
A small number of Ext-TEB molecules exhibit short-range diffusion on the Bi(111) surface. (**a**) High-resolution STM image of the Bi(111) surface (*V_s_* = 0.9 V, *I_t_* = 30 pA). (**b**) Structural models of the Ext-TEB molecule. The middle shows the planar Ext-TEB molecule before optimization. The left and right panels show the optimized Ext-TEB molecule with L- and R- torsions. (**c**–**f**) Dynamic process of Ext-TEB molecular cluster diffusion (*V_s_* = 1.8 V, *I_t_* = 30 pA). (**c**,**d**) The blue arrows indicate that there is an extra molecule at the corresponding position. (**d**,**e**) The green arrows indicate that there is an extra molecule at the corresponding position. (**e**,**f**) The yellow arrows indicate that the molecule positions have changed. The white arrows in the figure indicate the substrate crystal orientation, and the red arrows indicate the direction of the molecular main axis.

Alkynes, characterized by sp-hybridized ethynyl functional groups, exhibit high chemical reactivity and are of significant interest in the synthesis of low-dimensional organic interfaces. The self-assembly of alkynes on metal substrates often leads to the formation of well-ordered structures [17,18,19,20]. In particular, 1,3,5-Tris(4-ethynylphenyl) benzene (Ext-TEB, C_30_H_18_) is a rigid, C_3_-symmetric molecule featuring a central benzene core with three peripheral ethynylphenyl arms, as shown in Figure 1b. These terminal ethynyl groups enable its enhanced participation in intermolecular bonding [21,22]. On the Ag(111) surface, terminal alkynes of Ext-TEB molecules undergo coupling reactions to form organometallic honeycomb networks with shared edges [21,22,23,24]. In contrast, on the Cu(111) surface, the strong molecule–substrate interaction leads to the formation of close-packed supramolecular islands that remain stable at room temperature following annealing [25]. However, depositing Ext-TEB molecules on Cu(111) at low temperatures results in disordered structures. To date, few studies have leveraged substrate properties to induce chirality in otherwise achiral Ext-TEB molecules. Moreover, investigations of Ext-TEB molecules on semimetallic surfaces remain scarce.

Bismuth is a typical semimetal, characterized by low carrier concentration, small effective mass, and long mean free path [26]. The electronic structure of its bulk phase exhibits a slight overlap between the valence band and the conduction band, enabling Bi to possess both metallic and semiconductor properties [27,28,29]. The Bi(111) surface obtained through epitaxial growth exhibits a strong spin-orbit coupling effect, with its surface states being topologically protected and presenting non-trivial electronic characteristics [30,31,32]. Compared to metal substrates, the Bi(111) surface has a lower work function, which can reduce the interface charge injection barrier and help form a gentler interface dipole layer [33,34,35]. Moreover, the Bi(111) surface shows high chemical inertness and exhibits weak interactions with organic molecules, which suppresses interfacial chemical reactions and enables low-defect thin film via epitaxial growth or self-assembly of organic molecules [36]. These properties can be used to explore the mechanism underlying chirality formation in initially achiral Ext-TEB molecules.

In this study, we utilized LT-STM to investigate the chiral self-assembly and chiral separation of Ext-TEB molecules on the semimetallic Bi(111) surface. A small number of Ext-TEB molecules aggregated into clusters, with peripheral molecules displaying chiral characteristics and exhibiting short-range diffusive behavior. After film formation, the molecules formed basic units of a honeycomb structure without shared edges (HOSE). Subsequently, after annealing at 330 K, the HOSE structure transformed into a close-packed structure. Remarkably, both structural phases exhibited organizational and molecular chirality, accompanied by pronounced chiral separation.

## 2. Experimental Detail

The experiments were performed with an ultrahigh vacuum LT-STM system (Unisoku, Osaka, Japan) with a base pressure of about 2.0 × 10^−10^ Torr. A clean Si(111)-7 × 7 surface was prepared by continuous degassing at 850 K for over 7 h, followed by thermal flashing to 1500 K. Then, Bi atoms were deposited onto the Si(111)-7 × 7 surface by sublimation at 570 K. Following annealing to 370 K, the high-resolution STM image of the Bi(111) grown structure is shown in Figure 1a, with the lattice constant of *a*_0_ = 4.5 ± 0.2 Å. The structural models of the Ext-TEB molecule are depicted in Figure 1b: wherein the central portion represents a planar achiral Ext-TEB molecule before optimization, while the left and right panels illustrate the optimized Ext-TEB molecule with anticlockwise (L) and clockwise (R) torsions, respectively. Ext-TEB molecules (Macklin, Shanghai, China, purity >97%) were pre-degassed at 413 K in a quartz crucible before being sublimated at 393 K onto the Bi(111) surface at 80 K. The Ext-TEB coverage was controlled by the sublimation time. Here, we define one monolayer (ML) coverage of Ext-TEB as the amount of Ext-TEB molecules with the HOSE structure completely covering the Bi(111) surface. All STM images were acquired in constant current mode in a liquid nitrogen environment (77 K). The bias voltage was applied to the sample with respect to the tip. The *dI*/*dV* spectra were performed with a lock-in technique with a modulation voltage of 20 mV and frequency of 373 Hz at 77 K.

## 3. Results

### 3.1. Diffusion-Induced Chirality Switching

A small number of Ext-TEB molecules were deposited onto the Bi(111) surface at 80 K. Due to the weak molecule–substrate interaction with Bi(111), molecules located at the cluster edges are weakly confined and diffusive, offering an opportunity to probe the origin of chirality in Ext-TEB molecules. In Figure 1c, the cluster consists of 11 molecules, with the edge molecules exhibiting right-handed chirality, while the chirality of the central molecules cannot be distinguished. Continuous scanning of the same cluster revealed that molecular diffusion readily occurs during scanning. Figure 1c–f were obtained by consecutively scanning the same area, with the bright spot in the lower right corner serving as a marker. The comparison revealed that Ext-TEB molecules can diffuse from outside the scanned region into the scanned area, as indicated by the blue arrow in Figure 1d and the green arrow in Figure 1e, where they exhibit right-handed chirality at these positions. Within the scanning area, molecules can still diffuse around the cluster to adjacent positions, as shown by the yellow arrows in Figure 1e,f. A comparison before and after the movement reveals that, prior to moving, the molecule exhibits a rotation angle of 15° [we define it as an R-rotation of the molecular long axis with respect to the Bi substrate lattices, as indicated by the red and white arrows in Figure 1e], and they exhibit R- chirality. After moving, the molecular main axis aligns with the substrate lattice direction, as shown by the white arrow in Figure 1f, and the chirality disappears. This result indicates that the chirality of the Ext-TEB molecule originates from the mismatch between its molecular principal axis and the substrate lattice. Notably, this single-molecule chirality was not observed during growth on Cu(111) and Ag(111) surfaces [24,25]. This discrepancy is attributed to the unique electronic structure of the Bi surface; its extremely low carrier concentration, small effective mass, and very low density of states near the Fermi level result in a weak molecule-substrate interaction [36]. Under such weak coupling, intermolecular π-π stacking and van der Waals interactions become dominant, thereby inducing the rotation of phenyl rings in the molecular side chains and ultimately generating chirality. This observation is consistent with theoretical calculations conducted using the VASP 5.4.4 software, which show that the most stable configurations in the gas phase are precisely the L- and R- chirality molecules, as illustrated in Figure S1.

**Figure 2:**
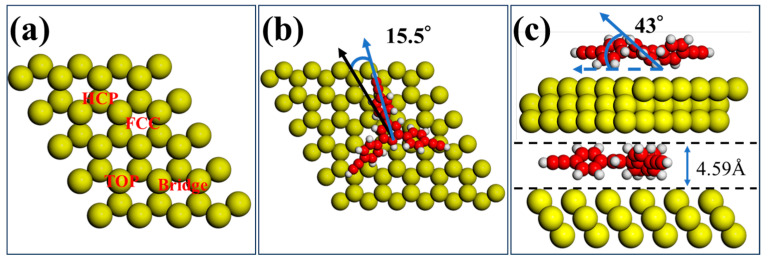
Schematic illustration of a single Ext-TEB molecule on the Bi(111) surface. (**a**) Top view of a single molecule at different adsorption sites on the Bi(111) surface. (**b**) Top view and (**c**) side view of a single Ext-TEB molecule at the optimal adsorption site on the Bi(111) surface, the black arrow indicates the crystal orientation of the substrate.

To investigate the stability of the Ext-TEB molecule on the Bi(111) surface, the center of a single R- chirality Ext-TEB molecule was placed at the top, bridge, hexagonal close-packed (HCP), and face-centered cubic (FCC) sites on a Bi(111)-6 × 6 supercell to calculate the adsorption energies, as shown in Figure 2a. The corresponding adsorption energies were −2.42 eV, −2.44 eV, −2.43 eV, and −2.42 eV, respectively. A comparison reveals that the Ext-TEB molecule is most stable at the bridge site, with a rotation angle of 15.5° between the molecular axis and the substrate crystal direction. The optimized structural model is shown in Figure 2b. The height difference between the molecular axis and the substrate is 4.59 Å, the torsion angle of the molecular side chain is 43°, as shown in Figure 2c. Thus, it can be determined that the chirality of Ext-TEB on the Bi(111) surface arises from the rotation of the benzene rings in the molecular side chains, induced by the mismatch between the molecular axis and the substrate crystal direction. This is similar to the origin of chirality observed for Tb_2_Pc_3_ molecules on the Pb(111) substrate surface [37].

### 3.2. Multilevel Chirality and Chiral Separation in HOSE Structures

**Figure 3:**
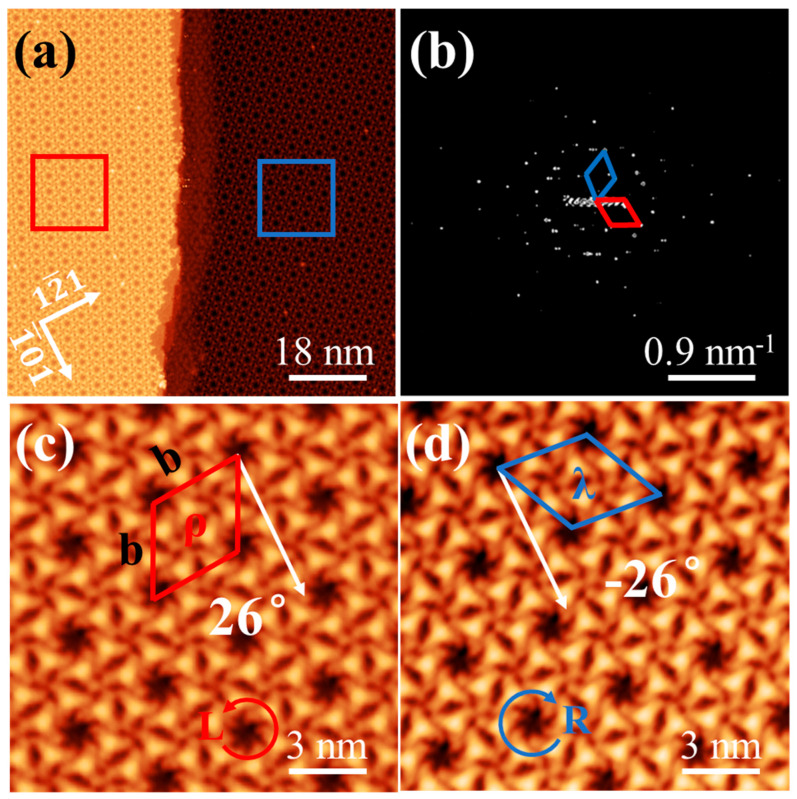
The monolayer of Ext-TEB exhibits a HOSE chiral structure. (**a**) Large-area STM image of the HOSE structures with different organizational chirality on two adjacent terraces (*V_s_* = 1.0 V, *I_t_* = 30 pA). (**b**) The corresponding fast Fourier transformation (FFT) image. (**c**) Enlarged view of the red box in (**a**), illustrating a *ρ*-domain of the L-HOSE structure showing the oblique angle of 26° relative to the [$\bar{1}01$] orientation (*V_s_* = 1.0 V, *I_t_* = 30 pA). (**d**) Enlarged view of the blue box in (**a**), showing a λ-domain of the R- HOSE structure viewed at an oblique angle of −26° relative to the [$\bar{1}01$] direction (*V_s_* = 1.0 V, *I_t_* = 30 pA).

Upon deposition of 1.0 ML at 80 K, Ext-TEB molecules formed a highly ordered HOSE structure on the Bi(111) surface, a behavior distinct from disordered structures on Cu(111) surface at low temperatures [25]. On Bi(111), we confirmed that a large-area, long-range ordered structure forms even at 80 K and can be observed at room temperature. This difference likely stems from a weaker interfacial interaction between Ext-TEB molecules and the Bi(111) surface compared to that with Cu(111). STM image in Figure 3a reveals a distinct chiral separation of the molecular domains above and below the step. The corresponding FFT analysis reveals that the lattice orientations above and below the step edges exhibit mirror symmetry, as shown in Figure 3b. In contrast, only a single enantiomorphic form exists within each monolayer, with the domain size being limited by the terrace width of the substrate, as shown in Figure S2. Statistical analysis of all STM images reveals an unequal distribution of enantiomorphic domains: 63% exhibit R-HOSE (composed of L- chiral molecules) and 37% exhibit L-HOSE (composed of R- chiral molecules). This non-equiprobable distribution, with a higher probability of L- chiral molecular domains, is consistent with the trend predicted by our gas-phase calculations. In Figure 3c, the Ext-TEB hexamers are aligned in the direction deviating 26° from the [$\bar{1}01$] direction of Bi(111) orientation, forming the right (*ρ*) enantiomorphic domain (organizational chirality). The lattice constant of HOSE structure is b = 35.1 ± 0.2 Å, corresponding to a high-order-commensurate (HOC) phase ($\sqrt{61}$ × $\sqrt{61}$) with packing density of 0.56 nm^−2^. The lattice chirality of *ρ*- domain can be described as a transformation matrix (5, 9, −4, 5), and six Ext-TEB molecules form an L- HOSE structure. The Ext-TEB molecules also form an R- HOSE structure, which appeared in the left (λ) enantiomorphic domains, the transformation matrix of λ- enantiomorphic domain is (5, −4, 9, 5), as shown in Figure 3d.

In both the *ρ*- and *λ*- domains, it is observed that within the hexamers, each molecule is rotated by 60° relative to its adjacent molecule. This phenomenon can be attributed to the synergistic effect of the sixfold symmetry of the Bi(111) substrate and intermolecular steric hindrance. Six Ext-TEB molecules form an L(R)- HOSE structure, achieving supramolecular chirality. The *ρ-* and *λ*- domains as well as the L- and R- HOSE structures have mirror symmetry with respect to the [$\bar{1}01$] direction of Bi(111) substrate.

**Figure 4:**
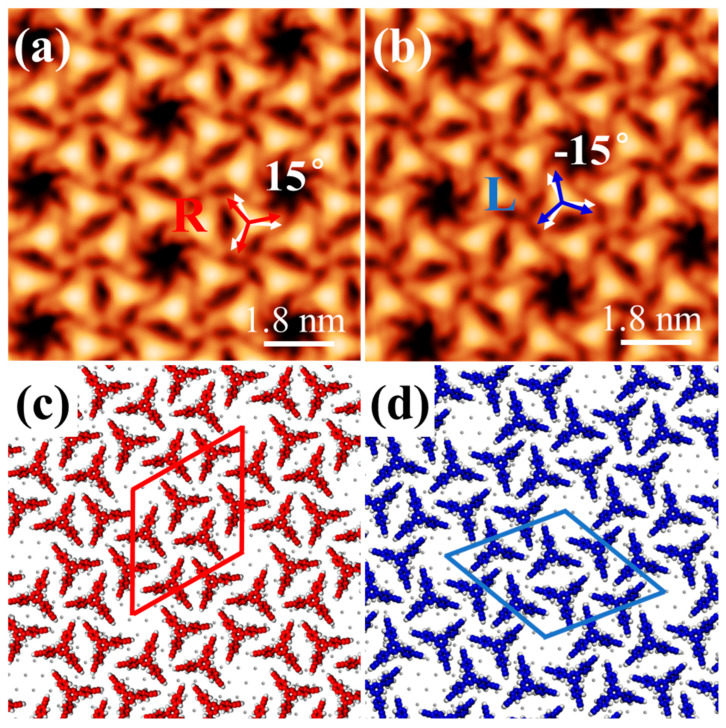
The chiral unit of the monolayer film consists of six molecules exhibiting identical chirality. (**a**) Within the ρ- domain, the individual molecules exhibit R- chirality and a rotation angle of 15°, the red arrows indicate the direction of the molecular main axis, and the white arrows denote the crystal orientation of the substrate (*V_s_* = 1.0 V, *I_t_* = 30 pA). (**b**) Within the λ- domain, individual molecules are found to have L- chirality and a rotation angle of −15° under identical measurement conditions, the blue arrows indicate the direction of the molecular main axis (*V_s_* = 1.0 V, *I_t_* = 30 pA). (**c**,**d**) are schematic structural models of *ρ*- and *λ*-domains.

Each Ext-TEB molecule features a triangular main skeleton with arc-shaped luminescent arms extending outward from its vertices. The asymmetric arrangement of these arms imparts conformational chirality to each molecule. This specific chirality arises from the fixed 15° offset between the primary molecular axis and the substrate lattice orientation, which generates two distinct chiral configurations. Within the *ρ*-domain, a molecule adopts an R- chiral configuration corresponding to a rotation angle of 15°, as shown in Figure 4a. Conversely, within the *λ*- domain, an L- chiral configuration is associated with a rotation angle of −15°, as shown in Figure 4b. The analysis reveals that the rotation of phenyl rings in the side chains, likely driven by interactions between terminal ethynyl groups [24], gives rise to the molecular chirality rather than electronic state effects.

In summary, the distinct rotational orientations of adjacent molecules within the two enantiomorphic domains, together with the differing alignment of individual molecules relative to the substrate lattice orientation, give rise to organizational chirality, supramolecular chirality, and single-molecule chirality in the monolayer film. The corresponding schematic models are shown in Figure 4c,d.

### 3.3. Annealing-Induced Transformation and Separation of Chiral Structures

**Figure 5:**
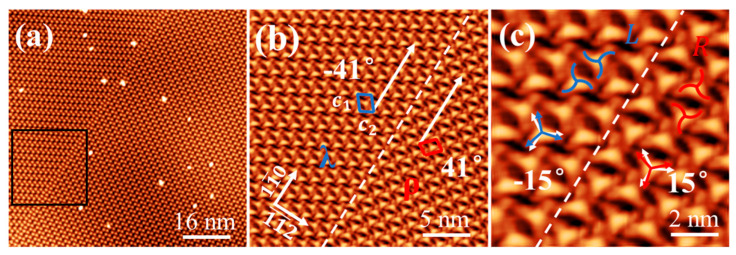
After annealing, the monolayer film forms a close-packed chiral structure. (**a**) Large-area close-packed structure formed after annealing (*V_s_* = 2.0 V, *I_t_* = 30 pA). (**b**) Chiral segregation in the close-packed structure: a *λ*- domain and a *ρ*- domain exhibiting oblique angles of ±41° relative to the [$1\bar{1}0$] direction (*V_s_* = 1 V, *I_t_* = 200 pA). (**c**) High-resolution STM image of (**c**), where the *λ*- domain consists of two L- chirality molecules as the unit cell, and the *ρ*- domain consists of two R- chiral molecules as the unit cell (*V_s_* = 1.0 V, *I_t_* = 200 pA).

Upon annealing to 330 K, part of the homochiral HOSE structure transforms into heterochiral molecular structures, as shown in Figure S3. This transition ultimately results in the formation of a large-area close-packed structure after 15 min of annealing, as shown in Figure 5a. This close-packed structure exhibits lattice chirality, manifested by two enantiomorphic domains (*λ*, *ρ*). The lattice constant of the Ext-TEB close-packed structure is *c*_1_ = 23.3 ± 0.2 Å, *c*_2_ = 14.2 ± 0.2 Å, θ = 101 ± 0.5°, corresponding to a HOC phase ($2\sqrt{7}$ × 3) with packing density of 0.62 nm^−2^. The lattice chirality of λ- domain can be described as a transformation matrix (3, 0, 2, 6), and the transformation matrix of *ρ*- enantiomorphic domain is (6, 2, 0, 3). The *λ*- and *ρ*- domains have a mirror symmetry with respect to the [$1\bar{1}0$] direction of Bi(111) substrate, and the direction of the domain boundary is consistent with the [$1\bar{1}0$] crystal orientation, as shown in Figure 5b. The close-packed structure consists of dimers as its unit cell. The molecular chirality is opposite in the two enantiomorphic domains: within the λ- domain, the individual molecules exhibit L- chirality, whereas in the *ρ*- domain, they display R- chirality, as shown in Figure 5c. Notably, the close-packed structure exhibits essentially equal probabilities of L- and R- chirality. This observation demonstrates that annealing drives the system to transform into a thermodynamically more stable close-packed structure. During this structural reconstruction, the weak molecule-substrate interaction on the Bi(111) surface provides sufficient freedom for molecular rearrangement, leading to the formation of a close-packed architecture with chiral separation. In contrast, Ext-TEB molecules on the Ag(111) surface exhibit only organizational chirality, without chiral separation, due to the relatively stronger molecule-substrate interaction [24]. This is in contrast to the growth of pentacene on Bi(111), where molecular thin films exhibit only supramolecular and organizational chirality [38]. This comparison demonstrates that the emergence of multilevel chirality in Ext-TEB molecules on Bi(111) arises from the crucial roles played by both the molecule and the substrate.

### 3.4. Bias-Dependent Electronic States in the HOSE Structure

**Figure 6:**
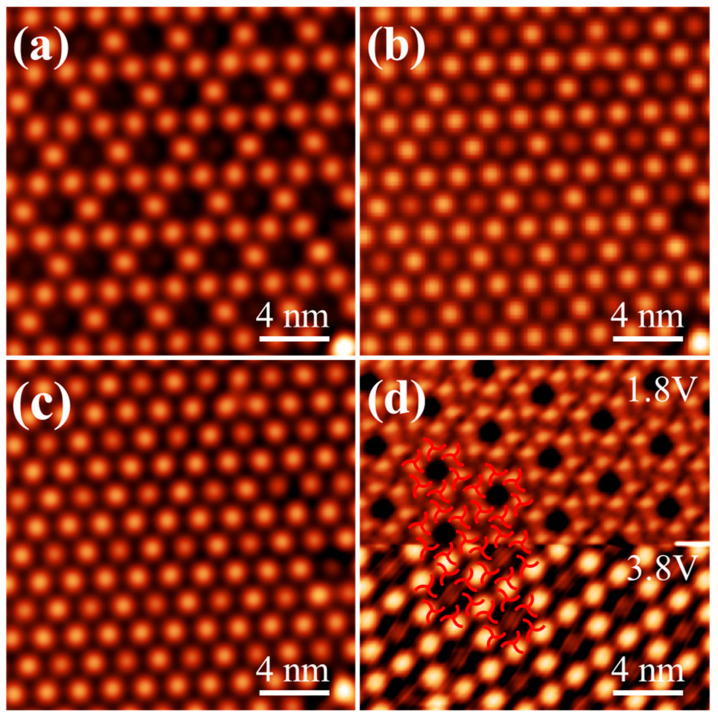
The STM image of the HOSE structure exhibits bias dependence. (**a**) At 3.8 V, the electronic state of the HOSE structure transitions into an edge-sharing configuration (*V_s_* = 3.8 V, *I_t_* = 20 pA). (**b**) At 4.0 V, dark spots appear at the centers of the edge-sharing configuration (*V_s_* = 4.0 V, *I_t_* = 20 pA). (**c**) At 4.2 V, all spots within the edge-sharing configuration exhibit nearly uniform brightness (*V_s_* = 4.2 V, *I_t_* = 20 pA). (**d**) Bias-dependent STM images of the HOSE structure, overlaid with molecular models (*V_s_* = 1.8, 3.8 V, *I_t_* = 20 pA).

Bias-dependent STM characterization of the HOSE structure reveals that at bias below 3.8 V, the molecular backbone exhibits a triangular configuration. When the bias reaches 3.8 V, each pair of molecules appears as a single bright spot, as shown in Figure 6a, and the electronic state of the HOSE structure transitions into an edge-sharing arrangement. Upon increasing the bias to 4.0 V, a dark spot emerges at the center of the edge-sharing configuration, exhibiting lower intensity than the surrounding molecular bright spots, as shown in Figure 6b. Further increasing the bias to 4.2 V leads to a gradual brightening of the central dark spot, eventually reaching an intensity comparable to that of the molecular bright spots, as shown in Figure 6c. In Figure 6d, the bias was dynamically switched from 1.8 V to 3.8 V during scanning, and the STM image clearly illustrates the apparent change of the molecules: at low bias, each molecule shows as a sharp spot superimposed on a triangular feature, while at high bias, each molecular dimer appears as a broad bright spot. This transition reflects that at low bias, the weak molecule-substrate interaction allows the molecules to retain their intrinsic clover-like conformation. At biases above 3.8 V, the unoccupied orbitals of the molecules hybridize with the surface states of Bi(111), forming new hybrid interface states. This hybridization modifies the spatial distribution of the local density of states, causing molecular pairs to appear as single bright spots in STM images, which manifests as the edge-sharing configuration.

## 4. Conclusions

In summary, we have investigated the chiral self-assembly and chiral separation of Ext-TEB molecules on Bi(111) using LT-STM. At 80 K, isolated molecules can diffuse and attach to the clusters, with a short-range thermal diffusion length. Increasing the coverage promotes the formation of a large-area, long-range ordered HOSE structure in the monolayer, which exhibits organizational, supramolecular, and molecular chirality. Subsequent annealing transforms the HOSE structure into a close-packed arrangement, retaining these chiral characteristics while inducing chiral separation. The observed chirality of Ext-TEB molecules is attributed to the mismatch between the orientation of the molecular backbone and the substrate lattice directions, highlighting the critical role of weak molecule–substrate interactions and intermolecular hydrogen bonding in the hierarchical expression of chirality. Additionally, the HOSE structure exhibits bias-dependence features, with its electronic state switching into an edge-sharing honeycomb configuration at high bias voltages. This phenomenon likely arises from the hybridization between the molecular unoccupied states and the Bi(111) surface states at highly excited states near the vacuum level, which enhances the local electronic states between adjacent molecules. These results contribute to a better understanding of the emergence of chirality from achiral molecules and pave the way for potential applications in enantioselective catalysis, molecular electronics, and the engineering of chiral surfaces.

## Data Availability

The data that support the findings of this study are available from the corresponding author upon reasonable request.

## Supplementary Materials

The following supporting information can be downloaded at: https://www.mdpi.com/article/10.3390/nano16070399/s1, see the supplementary material for additional details, including the theoretical calculation methods, the optimized structures and energies of gas-phase Ext-TEB molecules, the large-area monolayer HOSE structure, and the clusters formed after annealing at 330 K. References [39,40,41,42] are cited in the Supplementary Materials.
