# Supplementary material for: Chiral Self-Assembly and Chiral Separation of Ext-TEB Molecules on Bi(111)
Source: Nanomaterials (Basel). 2026 Mar 26;16(7):399. doi: 10.3390/nano16070399 (PMC13074591; doi:10.3390/nano16070399)
Supplement: Supplementary file 1 [file nanomaterials-16-00399-s001.zip › nanomaterials-4206177-supplementary.pdf]

Supporting Information

Chiral Self-Assembly and Chiral Separation of Ext-TEB Molecules on Bi(111)

Lei Liu 1, Zheng Wei 1,*, Min-Long Tao 2, Kai Sun 2, Ming-Xia Shi 3 and Jun-Zhong Wang 2,*

1 International Joint Laboratory for Light Alloys (MOE), College of Materials Science and Engineering, Chongqing University, Chongqing 400044, China; 20220901045@stu.cqu.edu.cn
2 School of Physical Science and Technology, Southwest University, Chongqing 400715, China; taotaole@swu.edu.cn (M.-L.T.); skqtt@swu.edu.cn (K.S.)
3 Key Laboratory for Electronic Materials, College of Electrical Engineering, Northwest Minzu University, Lanzhou 730030, China; 292229061@xbmu.edu.cn
* Correspondence: zheng_wei@cqu.edu.cn (Z.W.); jzwangcn@swu.edu.cn (J.-Z.W.)

Table of contents:

I. Computational Method

II. Optimized model of a single molecule under gas-phase conditions

III. Monolayer large-area HOSE structure with the same chirality

IV. Clusters with Mixed Chirality Formed after Annealing at 330 K

References

I. Computational Method

The theoretical calculations were performed using ab initio simulation. [1] The calculations were carried out using the VASP software package. The electron-ion interactions were described with the projector augmented wave (PAW) potentials and the electronic exchange-correlation energy was treated by the generalized-gradient approximation (GGA) of Perdew-Burke-Ernzerhof (PBE). [2,3] Van der Waals corrections were considered with the Grimme D3 method. [4] A 20 Å vacuum layer was utilized along the c direction normal to the surface. For the structure optimization, three atomic-layer Bi(111) substrate was adopted with bottom two layers fixed. The adsorption calculations were performed on Bi(111) surfaces modeled using a 6 × 6 supercell for single-molecule adsorption. The kinetic energy cutoff for the plane-wave expansion was set to 400 eV. The convergence criteria were set to 1.0×10^{-5} eV/atom for energy and 0.02 eV/Å for atomic forces.

To verify the stability of the Ext-TEB on the Bi(111) surface, the adsorption energies were estimated according to the following equation,

$$E_{\mathrm{ads}} = (E_{Ext\text{-}TEB@\ Bi(111)} - E_{Ext\text{-}TEB} - E_{Bi(111)}) \quad (1)$$

here $E_{Ext\text{-}TEB@\ Bi(111)}$ is the total energy of Ext-TEB on Bi(111) surface, $E_{Ext\text{-}TEB}$ and $E_{Bi(111)}$ are the energy of separated Ext-TEB and Bi(111) surface.

II. Optimized model of a single molecule under gas-phase conditions

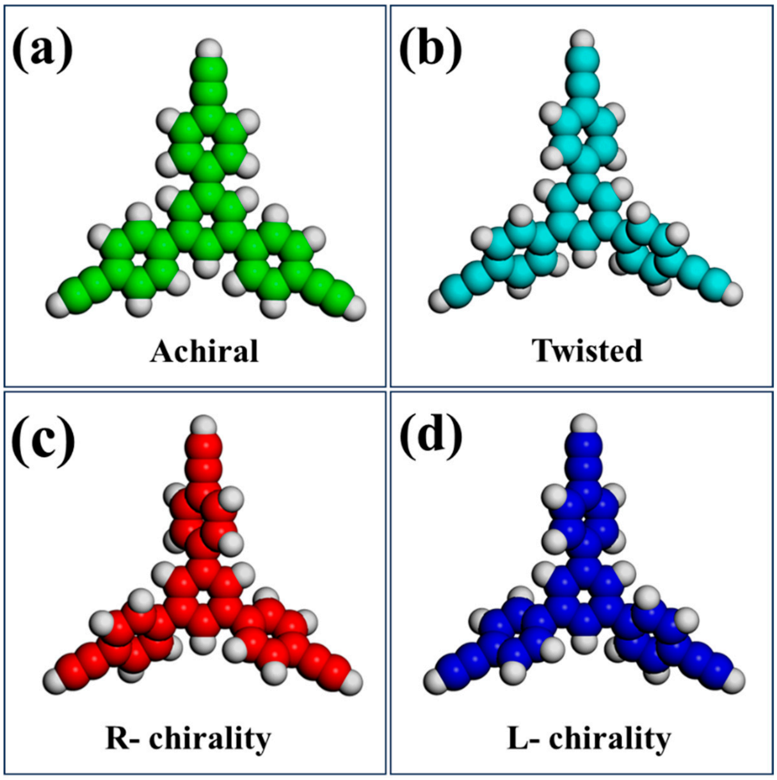

Figure S1. Optimized molecular models of Ext-TEB in the gas phase. (a) Planar molecular configuration, achiral; (b) Twisted molecules. (c) Molecular configuration with Right (R) chirality; (d) Molecular configuration with Left (L) chirality

We constructed four molecular configurations for DFT calculations, as illustrated in Fig. S1: (a) a planar molecule in which the side chains do not rotate; (b) a twisted molecule in which two phenyl rings in the side chains rotate clockwise by 45° and one phenyl ring rotates counterclockwise by 45°; (c) a right chirality molecule in which all three phenyl rings in the side chains rotate counterclockwise by 45°; (d) a left chirality molecule in which all three phenyl rings rotate clockwise by 45°. Geometry optimization using DFT calculations revealed that the torsion angle of the side chains in the left-handed and right-handed molecules is 40°. The calculated energies for the planar, twisted, right-handed, and left-handed molecules are -331.079 eV, -331.317 eV, -331.320 eV, and -331.328 eV, respectively. These results indicate that, under gas-phase

conditions, the left-handed and right-handed configurations represent the most stable molecular conformations.

III. Monolayer large-area HOSE structure with the same chirality

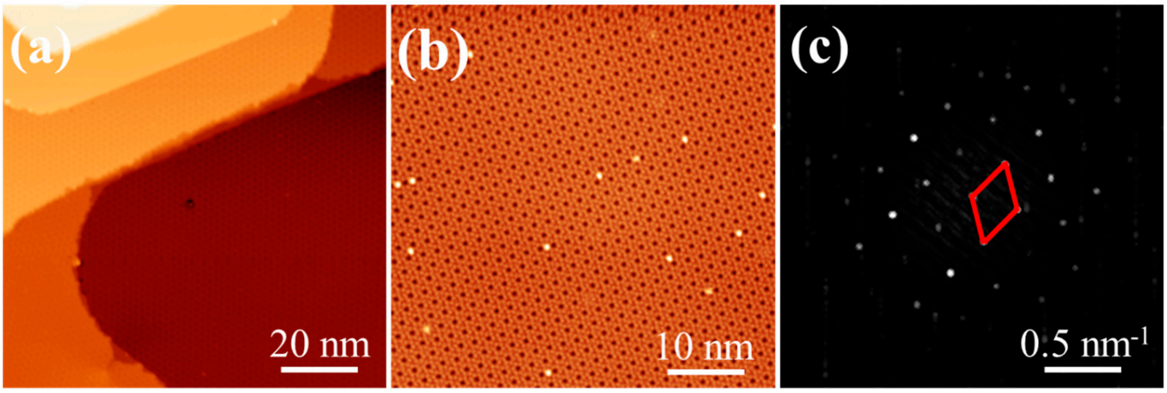

Figure S2. The HOSE structure of the Ext-TEB monolayer. (a) STM image showing a large-area view of the monolayer HOSE structure (V_s = 2.5 V, I_t = 30 pA). (b) A magnified view of the HOSE structure (V_s = 2.0 V, I_t = 30 pA). (c) is FFT image of panel (b).

IV. Clusters with Mixed Chirality Formed after Annealing at 330 K

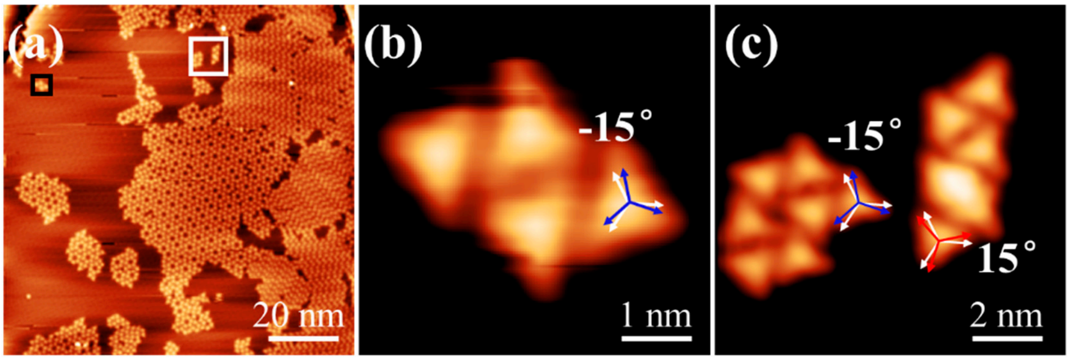

Figure S3. Upon annealing the monolayer film of Ext-TEB molecules at 330 K, molecules within the same layer exhibit both L- and R- chirality. (a) Intermediate stage of the structural transition from the HOSE structure to a close-packed arrangement. (V_s = 1.9 V, I_t = 30 pA). (b) Magnified view of the black square in panel (a), Ext-TEB molecules form a tetramer on the Bi(111) surface. The blue arrows in the figure indicate the direction of the molecular principal axis, which forms an angle of -15° with respect to the substrate crystal orientation. (V_s = 1.9 V, I_t = 30 pA). (c) Magnified view of the white square in panel (a), Ext-TEB molecules form two heptamers on the Bi(111) surface. The red (blue) arrows in the figure indicate the direction of the molecular principal axis, which

forms an angle of ±15° with the substrate crystal orientation (V_s = 1.8 V, I_t = 30 pA).

References

1. Troullier, N.; Martins, J. L. Efficient pseudopotentials for plane-wave calculations. Phys. Rev. B 1991, 43, 1993-2006.
2. Blöchl, P. E. Projector augmented-wave method. Phys. Rev. B 1994, 50, 17953-17979.
3. J. P. Perdew; K. Burke; M. Ernzerhof. Generalized gradient approximation made simple. Phys. Rev. Lett. 1996, 77, 3865.
4. Grimme, S.; Antony, J.; Ehrlich, S.; Krieg, H. A consistent and accurate ab initio parametrization of density functional dispersion correction (DFT-D) for the 94 elements H-Pu. J. Chem. Phys. 2010, 132, 154104
